# The complete chloroplast genome of *Schizostachyum dumetorum* var. *xinwuense* (Bambusoideae: Poaceae)

**DOI:** 10.1080/23802359.2021.1889410

**Published:** 2021-03-18

**Authors:** Yu Zhao-yan, Tan Yu-shan, Zhou Jie, Li Jia-jia, Guo Qi-rong

**Affiliations:** a Co-Innovation Center for Sustainable Forestry in Southern China, Nanjing Forestry University, Nanjing, China; b International Center of Bamboo and Rattan, Beijing, China

**Keywords:** *Schizostachyum dumetorum* var. *xinwuense*, chloroplast genome, phylogenetic analysis, sequencing

## Abstract

*Schizostachyum dumetorum* var. *xinwuense* (T.H.Wen & J.Y.Chin) N.H.Xia is an arborescent bamboo that is native to Jiangxi, China. The bamboo culm wall of this species is extremely thin, which has high economic value and ecological benefits. Here, we assembled the complete chloroplast genome of *S. dumetorum* var. *xinwuense* using Illumina pair-end sequencing data in this work. The total genome size of *S. dumetorum* var. *xinwuense* was 139,230 bp in length, containing a large single-copy (LSC) region of 82,876 bp, a small single-copy (SSC) region of 12,877 bp, and a pair of inverted repeat (IR) regions of 21,777 bp. The overall GC content of the genome was 38.87%, and the corresponding GC values of the LSC, SSC, and IR regions were 36.98%, 32.83%, and 44.17%, respectively. The genome contained a total of 112 genes, including 75 protein-coding, 30 tRNA, and seven rRNA. Phylogenetic analysis of *S. dumetorum* var. *xinwuense* positioned it in a strongly supported clade with *Arundinaria appalachiana*. These data show the phylogenetic location of *Schizostachyum dumetorum* var. *xinwuense* within the family of the Bambusoideae and contribute to the biodiversity and systematics of the Bambusoideae.

*Schizostachyum dumetorum* var. *xinwuense* is a large, thicket forming bamboo that consists of two varieties, var. *dumetorum* and var. *xinwuense*. The two species differ in their culm sheaths. *S. dumetorum* var. *xinwuense* displays glabrous sheaths, while those of *S. dumetorum* var. *xinwuense* are hairy. Different from ordinary bamboos, the wall of *S. dumetorum* var. *xinwuense* is extremely thin, brittle, and has remarkable thin walled (Wen 1982). In addition, the height of *S. dumetorum* var. *xinwuense* can reach 8 m with leaves that are lanceolate to narrow lanceolate. This variety is endemic to China and cultivated in Jiangxi as an ornamental (Editorial Committee of Flora of Chinese Academy of Sciences 2006). In this study, the complete chloroplast (cp) genome of *S. dumetorum* var. *xinwuense* was analyzed to document the genetic biodiversity of the species and to determine its phylogenetic relationship to other Bambusoideae.

The fresh leaves of *S. dumetorum* var. *xinwuense* were collected from the experimental bamboo forest (113.1124063°E, 28.2698183°N, 44.9 m above sea level) in Lukou Town, Changsha County, Hunan Province, China. The voucher specimen deposited in the college of forestry, Nanjing Forestry University (NJFU-2020796, Professor Guo, qrguo@njfu.edu.cn). The total genomic DNA was extracted and sequenced using the Illumina HiSeq 2500 platform. The 150 bp paired-end sequencing generated approximately 68.5 GB of raw data. We used the GetOrganelle software to assemble the complete cp genome (Jin et al. 2020). The genome annotated with the program Geneious R8 (Biomatters Ltd, Auckland, New Zealand) by comparing the cp genome of *S. dumetorum* var. *xinwuense* to the reference genome of *Phyllostachys edulis* (HQ337796) (Tillich et al. 2017). The tRNA genes were further confirmed through online tRNAscan-SE web servers (Schattner et al. 2005). The cp genome data of *S. dumetorum* var. *xinwuense* were uploaded to GenBank (https://www.ncbi.nlm.nih.gov/genbank/), and its number was LC590825. The genome alignment was achieved using MAFFT (Katoh and Standley 2013). The best-fitted model was selected by ModelFinder (Kalyaanamoorthy et al. 2017).

The cp genome of *S. dumetorum* var. *xinwuense* is quadripartite and 139,230 bp in length. It consists of a large single-copy (LSC) region of 82,876 bp and a small single-copy (SSC) region of 12,877 bp, separated by two inverted repeat (IR) regions of 21,777 bp, respectively. The GC content of the total genome was 38.87%, whereas the IR region had a higher GC content (44.17%) than LSC (36.98%) and SSC (32.83%). The cp genome encoded 112 genes, including 75 protein-coding, 30 tRNA, and seven rRNA genes.

The phylogenetic analysis strongly supported a close relationship between *S. dumetorum* var. *xinwuense* and *Arundinaria appalachiana* (Figure 1). The two species are positioned in a sister clade to *A. fargesii*. The cp genome of *S. dumetorum* var. *xinwuense* will provide useful genetic information for further study on genetic diversity and conservation of bamboo species.

**Figure 1.**
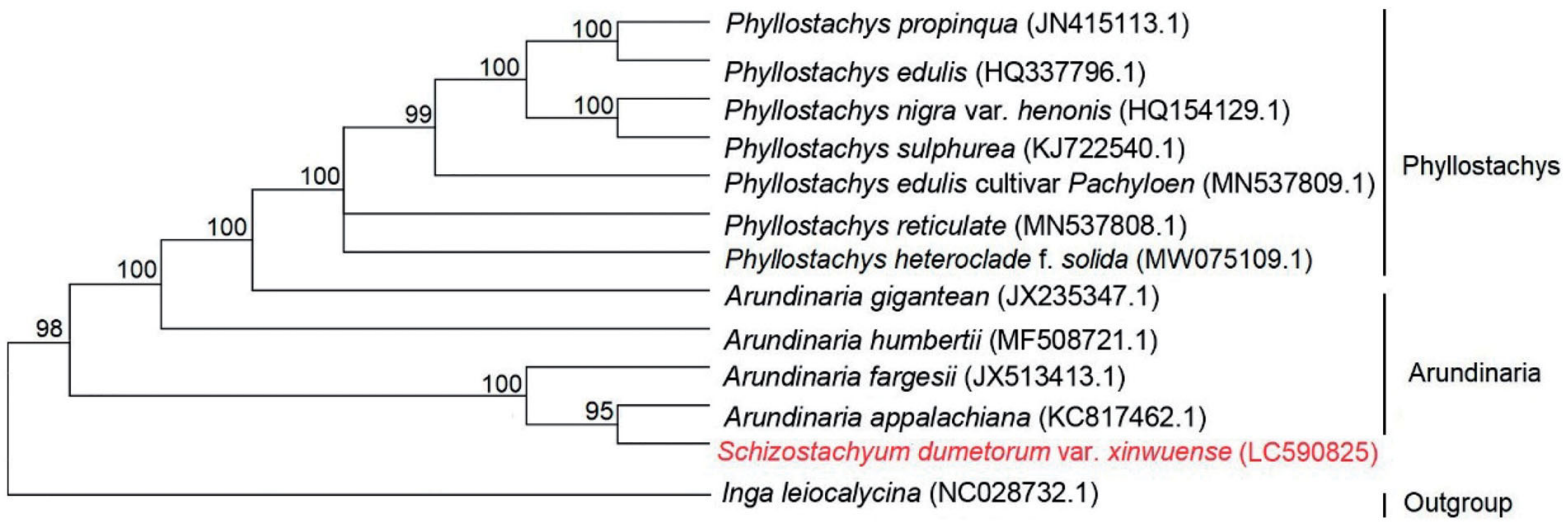
Phylogenetic relationships among *Schizostachyum dumetorum* var. *xinwuense* and 12 complete chloroplast genomes of bamboo species. Bootstrap support values are given at the nodes.

## Data Availability

The genome sequence data that support the findings of this study are openly available in GenBank at https://www.ncbi.nlm.nih.gov/genbank/ under the accession no. LC590825. The associated BioProject, SRA, and Bio-Sample numbers are PRJNA642921, SRP269283, and SAMN15401931, respectively, in NCBI.
